# A normalized dataset of 1821 cortical and subcortical functional responses collected during direct electrical stimulation in patients undergoing awake brain surgery

**DOI:** 10.1016/j.dib.2019.104892

**Published:** 2019-12-05

**Authors:** Silvio Sarubbo, Matthew Tate, Alessandro De Benedictis, Stefano Merler, Sylvie Moritz-Gasser, Guillaume Herbet, Hugues Duffau

**Affiliations:** a Division of Neurosurgery, Structural and Functional Connectivity Lab Project, Azienda Provinciale per i Servizi Sanitari (APSS), 9 Largo Medaglie d’Oro, 38122, Trento, Italy; b Departments of Neurosurgery and Neurology, Northwestern University, Feinberg School of Medicine, 420 E Superior St, 60611, Chicago, IL, USA; c Neurosurgery Unit, Department of Neuroscience and Neurorehabilitation, Bambino Gesù Children's Hospital IRCCS, 4 Piazza Sant'Onofrio, 00165, Rome, Italy; d Bruno Kessler Foundation (FBK), Trento, Italy; e Department of Neurosurgery, Gui de Chauliac Hospital, Montpellier University Medical Center, 80 Avenue Augustin Fliche, Montpellier, France; f National Institute for Health and Medical Research (INSERM), U1051, Team “Plasticity of the Central Nervous System, Human Stem Cells and Glial Tumors”, Institute for Neurosciences of Montpellier, Montpellier University Medical Center, 80 Av Augustin Fliche, Montpellier, France

**Keywords:** Atlas, Brain functions, Brain mapping, Direct electrical stimulation, White matter

## Abstract

In this data article, we provide the dataset which served as the basis for our related research article “Mapping critical cortical hubs and white matter pathways by direct electrical stimulation: an original functional atlas of the human brain” [1], which represents the first probabilistic cortical and subcortical atlas of critical structures mediating human brain functions based on direct electrical stimulation (DES) in patients undergoing awake brain surgery. 1162 cortical and 659 subcortical DES-derived responses were recorded during testing of 16 functional domains in 256 patients undergoing awake surgery. Normalized [Montreal Neurological Institute (MNI) 152] spatial coordinates for cortical and subcortical responses, and probabilistic heat maps for each functional domain, were computed using methods previously developed by our group [2,3]. Source data, including the MNI-normalized coordinates of all 1821 DES-derived cortical and subcortical data points, and multi-planar (MNI-152, T1 1mm) videos showing the probabilistic distribution of each functional domain are provided. This novel dataset can improve and refine our understanding about the functional anatomy of critical brain networks, and these data are made available for medical and neuroscience applications.

**Table:** Specifications Table

Subject	Behavioral Neuroscience
Specific subject area	Functional human brain mapping data
Type of data	Tables (2) Videos (16)
How data were acquired	Direct electrical stimulation of the cortical surface and subcortical white matter in patients undergoing awake brain surgery.
Data format	Raw data: normalized MNI coordinates (x,y,z) for all 1821 stimulation points. Analysed: videos of the spatial cortico-subcortical probabilistic distributions for 16 key brain functions on axial, sagittal and coronal T1 (MN1-152, 1 mm) sequences.
Parameters for data collection	Cortical or subcortical sites for which promotion or interruption of function were observed during intra-operative direct electrical stimulation were collected.
Description of data collection	Intra-operative photographs were taken for collection of positive cortical and subcortical stimulation sites. Data were then plotted onto standardized MNI-152 template (T1, 1 mm) brain for each patient.
Data source location	Department of Neurosurgery, Gui de Chauliac Hospital, Montpellier University Medical Center, 80 Avenue Augustin Fliche, Montpellier (France) Division of Neurosurgery, Structural and Functional Connectivity Lab Project, Azienda Provinciale per i Servizi Sanitari (APSS), 9 Largo Medaglie d’Oro, 38122 Trento (Italy)
Data accessibility	With the article.
Related research article	S. Sarubbo, M. Tate, A. De Benedictis, S. Merler, S. Moritz-Gasser, G. Herbet, H. Duffau, Mapping critical cortical hubs and white matter pathways by direct electrical stimulation: an original functional atlas of the human brain, Neuroimage. 205 (2020) 116237. https://doi.org/10.1016/j.neuroimage.2019.116237.

**Table:** 

**Value of the Data** • Here we present the first integrated and comprehensive cortical-subcortical atlas of structures essential for humans' neural functions based on highly-specific direct electrical stimulation-based mapping during real-time neuropsychological testing in a very large dataset (>250 patients) and with an unprecedented number of functional responses (>1800). • These data provide a template of critical brain nodes, at both the cortical and subcortical levels, for major functional categories, which can be utilized to better understand the relationships between brain injuries and subsequent functional deficits, an issue of importance to neurologists, neurosurgeons, psychologists, and rehabilitation physicians. • This novel and unique atlas can serve as a reliable and complementary tool for future multi-modal modeling and analyses exploring the structure and function of brain processing in humans.

## 1 Data

Location of main functional hubs of brain networks at the cortical level remains an open challenge for neuroscientists involved into connectome exploration as well as neurosurgeons aiming to achieve safe yet meaningful brain tumor resections. In addition, the identification network connections at the level of local and distant white matter pathways, has proved even more challenging, given that no non-invasive neuroimaging techniques exist that are able to provide information about the functional processing at the subcortical level.

In 2015 our Group published the first functional atlas of human white matter based on direct electrical stimulation (DES) during awake surgery procedures for resection of low-grade gliomas (LGGs) [3,4]. Recently we reported the unique and sole atlas integrating an unprecedented number (1821) of subcortical (659) and cortical (1162) functional responses collected in a large series of 256 patients, among 16 functional domains [1]. Each different category of functional response (semantic paraphasia, movement, sensation, etc.) is associated with a network (or sub-network) explored (e.g. movement arrest is associated with motor planning network).

In addition, the atlas includes a probability distribution of all functional responses collected, at both the cortical and subcortical levels, based on a multinomial statistical analysis of the frequency in eliciting each response. The probabilistic maps for each network were computed in 1mm Montreal Neurological Institute (MNI) space, and are presented in this report as separate videos for each cortical-subcortical functional distribution (videos 1–16).

Supplementary video related to this article can be found at https://doi.org/10.1016/j.dib.2019.104892.

The following are the supplementary data related to this article:

Video 1 *Motor.* In this video the probabilistic cortico-subcortical distribution of motor functional responses is reported in the axial, sagittal, and coronal planes of the MNI-152 template brain (T1-weighted sequence, 1mm resolution) as a color heat map representing cohort-level confidence intervals. Video 1

Video 2 *Somatosensory.* In this video the probabilistic cortico-subcortical distribution of somatosensory functional responses is reported in the axial, sagittal, and coronal planes of the MNI-152 template brain (T1-weighted sequence, 1mm resolution) as a color heat map representing cohort-level confidence intervals. Video 2

Video 3 *Motor Control.* In this video the probabilistic cortico-subcortical distribution of functional responses related to motor control is reported in the axial, sagittal, and coronal planes of the MNI-152 template brain (T1-weighted sequence, 1mm resolution) as a color heat map representing cohort-level confidence intervals. Video 3

Video 4 *Language and motor planning.* In this video the probabilistic cortico-subcortical distribution of functional responses related to language and motor planning is reported in the axial, sagittal, and coronal planes of the MNI-152 template brain (T1-weighted sequence, 1mm resolution) as a color heat map representing cohort-level confidence intervals. Video 4

Video 5 *Eyes movement control.* In this video the probabilistic cortico-subcortical distribution of functional responses related to eyes movement control [5] is reported in the axial, sagittal, and coronal planes of the MNI-152 template brain (T1-weighted sequence, 1mm resolution) as a color heat map representing cohort-level confidence intervals. Video 5

Video 6 *Speech output.* In this video the probabilistic cortico-subcortical distribution of functional responses related to speech output [2] is reported in the axial, sagittal, and coronal planes of the MNI-152 template brain (T1-weighted sequence, 1mm resolution) as a color heat map representing cohort-level confidence intervals. Video 6

Video 7 *Speech articulation.* In this video the probabilistic cortico-subcortical distribution of functional responses related to speech articulatory network [6] is reported in the axial, sagittal, and coronal planes of the MNI-152 template brain (T1-weighted sequence, 1mm resolution) as a color heat map representing cohort-level confidence intervals. Video 7

Video 8 *Anomia.* In this video the probabilistic cortico-subcortical distribution of anomia is reported in the axial, sagittal, and coronal planes of the MNI-152 template brain (T1-weighted sequence, 1mm resolution) as a color heat map representing cohort-level confidence intervals. Video 8

Video 9 *Semantic.* In this video the probabilistic cortico-subcortical distribution of semantic functional responses is reported in the axial, sagittal, and coronal planes of the MNI-152 template brain (T1-weighted sequence, 1mm resolution) as a color heat map representing cohort-level confidence intervals. Video 9

Video 10 *Non-verbal comprehension disorders.* In this video the probabilistic cortico-subcortical distribution of non-verbal comprehension disorders [7] is reported in the axial, sagittal, and coronal planes of the MNI-152 template brain (T1-weighted sequence, 1mm resolution) as a color heat map representing cohort-level confidence intervals. Video 10

Video 11 *Phonologic.* In this video the probabilistic cortico-subcortical distribution of phonological functional responses is reported in the axial, sagittal, and coronal planes of the MNI-152 template brain (T1-weighted sequence, 1mm resolution) as a color heat map representing cohort-level confidence intervals. Video 11

Video 12 *Visual.* In this video the probabilistic cortico-subcortical distribution of positive visual functional responses [8] is reported in the axial, sagittal, and coronal planes of the MNI-152 template brain (T1-weighted sequence, 1mm resolution) as a color heat map representing cohort-level confidence intervals. Video 12

Video 13 *Reading.* In this video the probabilistic cortico-subcortical distribution of reading functional responses [4,9] is reported in the axial, sagittal, and coronal planes of the MNI-152 template brain (T1-weighted sequence, 1mm resolution) as a color heat map representing cohort-level confidence intervals. Video 13

Video 14 *Spatial perception.* In this video the probabilistic cortico-subcortical distribution of spatial perception [10] disorders is reported in the axial, sagittal, and coronal planes of the MNI-152 template brain (T1-weighted sequence, 1mm resolution) as a color heat map representing cohort-level confidence intervals. Video 14

Video 15 *Mentalizing.* In this video the probabilistic cortico-subcortical distribution of mentalizing disorders [11] is reported in the axial, sagittal, and coronal planes of the MNI-152 template brain (T1-weighted sequence, 1mm resolution) as a color heat map representing cohort-level confidence intervals. Video 15

Video 16 *Acoustic.* In this video the probabilistic cortico-subcortical distribution of acoustic functional responses [[12], [13], [14]] is reported in the axial, sagittal, and coronal planes of the MNI-152 template brain (T1-weighted sequence, 1mm resolution) as a color heat map representing cohort-level confidence intervals. Video 16

The value of this unique dataset for improving surgical and clinical practice relates to the uniqueness of functional data obtained from in-vivo brain mapping in a large and homogeneous cohort of patients. In addition to the clinical impact of these data, the exact location of large number of cortical and subcortical functional sites will be useful for integration into multimodal neuroscience studies focused on resolving the complex structural and functional networks that constitute human brain processing.

For this purpose, we make available in this report the full list of normalized MNI-152 spatial coordinates of each of the 1821 functional responses collected for the cortical-subcortical version of our functional brain atlas in two separate tables (Table 1 for cortical functional responses; Table 2 for subcortical functional responses).

## 2 Experimental design, material and methods

All the functional responses were collected during asleep-awake-asleep surgery procedures with bipolar stimulation (frequency: 60Hz; pulse duration: 1 ms; intensity range: 2–4 mA) during execution of dedicated functional tasks, as previously reported [3,4]. All the 256 patients (mean age: 38.7 years; M:135, F:121; 85.1% right-handers, 9.4% left-handers, 5.5 ambidextrous) were affected by LGGs (60.6% in the left hemisphere, 39.4% in the right hemisphere). No patients had neurological deficits before surgery. Intraoperatively, functional sites were noted with numeric tags and then normalized coordinates tabulated based on a combination of intraoperative photographs along with post-operative 1-mm axial/sagittal/coronal T1-weighted MRI reconstructions by two expert anatomists (S.S. and M.T.).

## Appendix A Supplementary data

Multimedia component 1 Table 1. *Cortical functions.* In this table the full list of functional responses elicited during cortical direct electrical stimulation are provided along with the respective MNI coordinates. Table 2. *Subcortical functions.* In this table the full list of functional responses elicited during subcortical direct electrical stimulation are provided along with the respective MNI coordinates. Multimedia component 1
