# Supplementary material for: A normalized dataset of 1821 cortical and subcortical functional responses collected during direct electrical stimulation in patients undergoing awake brain surgery
Source: Data Brief. 2019 Dec 5;28:104892. doi: 10.1016/j.dib.2019.104892 (PMC6921148; doi:10.1016/j.dib.2019.104892)
Supplement: Multimedia component 1 — Table 1. Cortical functions. In this table the full list of functional responses elicited during cortical direct electrical stimulation are provided along with the respective MNI coordinates. Table 2. Subcortical functions. In this table the full list of functional responses elicited during subcortical direct electrical stimulation are provided along with the respective MNI coordinates. [file mmc1.docx]

**Table 1**

In this table the full list of functional responses elicited during **cortical** direct electrical stimulation are provided along with the respective MNI coordinates.

**Table 2**

In this table the full list of functional responses elicited during **subcortical** direct electrical stimulation are provided along with the respective MNI coordinates.
